# Supplementary material for: Homemade Nucleic Acid Preservation Buffer Proves Effective in Preserving the Equine Faecal Microbiota over Time at Ambient Temperatures
Source: Animals (Basel). 2023 Oct 5;13(19):3107. doi: 10.3390/ani13193107 (PMC10572018; doi:10.3390/ani13193107)
Supplement: Supplementary file 1 [file animals-13-03107-s001.zip › animals-2562236-supplementary.pdf]

Homemade nucleic acid preservation buffer proves effective in preserving the equine faecal microbiota over time at ambient temperatures

Ashley B. Ward [1,2]*, Patricia A. Harris[3], Caroline M. Argo[1], Christine Watson[1], Madalina Neacsu[2], Wendy R. Russell[2], Antonio Ribeiro[4], Elaina Collie-Duguid[4], Zeynab Heidari[4], Philippa K. Morrison[1]

[1] Scotland's Rural College, Aberdeen, UK
[2] University of Aberdeen, The Rowett Institute, Foresterhill, Aberdeen, UK
[3] Equine Studies Group, Waltham Petcare Science Institute, Leicestershire, UK
[4] Centre for Genome-Enabled Biology and Medicine, University of Aberdeen, King's College, Aberdeen, UK
School of Medicine Medical Sciences and Nutrition, University of Aberdeen, Foresterhill, Aberdeen, UK

* Correspondence: Ashley.Ward@sruc.ac.uk

DNA Extraction Protocol

Reference: Yu Z, Morrison M. 2004. Improved extraction of PCR-quality community DNA from digesta and fecal samples. Biotechniques 36: 808–812

Consumables required

- (N=X) pre-sterilised 2ml screw cap tubes containing 0.1g 0.5mm and 0.3g of 0.1mm zirconia beads – labelled
- (N=X) fresh sterile 2ml Eppendorf tubes – labelled
- (N=X * 2) fresh sterile 1.5 ml Eppendorf tubes – labelled
- (N=X) fresh sterile 1.5 ml Eppendorf tubes – labelled
- (N=X) QIAMP DNA collection columns
- (N=X) fresh collection tubes
- (N=X) * 3 fresh sterile 1.5 ml Eppendorf tubes – labelled for storage
- 1000 ul pipette tips
- 200 ul pipette tips
- 2 ul pipette tips
- 6 tube racks
- Storage boxes

Reagents and buffers

- Lysis buffer (500 uM NaCl, 50mM EDTA, 50 mM Tris-Hcl at pH 8, 4% SDS)
- 10M ammonium acetate
- TE buffer
- DNase free RNase (QIAGEN)
- Proteinase K (QIAGEN)
- Buffer AL (QIAGEN)
- Buffer AW1 (QIAGEN)
- Buffer AW2 (QIAGEN)
- Buffer AE (QIAGEN)

Preparation

1. Heat water bath to 70°C
2. Heat lysis buffer and syringe filter required volume into a beaker. Keep beaker warm.
3. Place ammonium acetate into freezer at -20°C
4. Bring isopropanol to room temperature
5. Place 70% ethanol into freezer at -20 °C

Method

Cell lysis

6. Transfer 0.25g of sample into a 2ml screw cap tube containing beads.

Make up tubes with beads in advance and sterilise in autoclave before using. This will damage some of the caps so need to be careful and replace caps as needed, or use tubes with seals resistant to heat

7. Add 1 ml lysis buffer

The SDS in the lysis buffer will come out of solution quite easily. Needs heated to 70°C and then kept quite warm for the next few steps. Beakers containing the lysis buffer can be kept in hot water within another beaker in a bain-marie style.

Lysis buffer was filter sterilised before use, due to the high detergent concentration, autoclaving not appropriate

8. Homogenise for 3 minutes at maximum speed

We used Disruptor Genie, Scientific Industries, Genie SI-DD38 Digital Disruptor Shaker, 120 VAC

9. Incubate at 70°C for 15 minutes, with manual shaking every 5 minutes

In water bath, care must be taken with the lids otherwise the sample will foam up and come out

10. Centrifuge at 16 000 x g for 5 minutes at 4 °C

11. Transfer 400 ul of the supernatant into a clean 2 ml Eppendorf and store on ice

12. Add 300 ul of lysis buffer to the original sample to repeat the bead beating step

13. Repeat steps 8, 9 and 10.

14. Transfer 100 ul of supernatant **into the Eppendorf – pooling the two samples together and discarding the pelleted matter**

DNA precipitation

15. Add 260 ul of ice cold 10M ammonium acetate and mix by vortexing briefly

This is a concentrated salt. DNA precipitates in the presence of 0.5M – 2.5 M salt and alcohol. If you have 500 ul of sample from the previous 2 steps, adding 260ul of 10M will take the concentration down to 3.4M

Ammonium acetate releases harmful aerosols when heated, so is filter sterilised, not autoclaved, before use.

16. Incubate on ice for 5 minutes

17. Centrifuge for 10 minutes at 4°C 16000 x g

18. Transfer the supernatant into 2 fresh 1.5 ml tubes

19. Add 1 volume of room temperature isopropanol and mix well (inverting the tubes several times)

20. Incubate on ice for 30 minutes.

The protocol by Yu and Morrison (2004) indicates to incubate on ice, however literature suggests that shorter incubations at room temperature are more appropriate for isopropanol. This should be tested under specific lab conditions.

21. Centrifuge the samples at 16000 x g for 15 minutes

22. Remove the supernatant by decanting and aspirating

Some of the samples will not have pelleted depending on the buffer the sample was initially stored in. In this case, remove the obvious supernatant and continue as below.

23. Add 1 ml of ice cold 70% ethanol and mix well

The purpose of the 70% ethanol is to remove any contaminating salts, but also ensure that the DNA remains in precipitated form – i.e the pellet. Ensure that you get all sides of the tube covered in ethanol and give a quick pulse on the vortex. If you vortex too vigorously, you risk shearing the DNA. If you don't vortex at all, you risk leaving salts coming through to the next step.

There were some difficulties with extracting the DNA from the NAP buffer samples at this stage if the buffer hadn't been fully removed at the initial stages. In those cases, the DNA precipitation step was repeated with 70% ice cold ethanol.

24. Centrifuge at 16000 xg for 5 minutes , 4°C

25. Repeat steps 22, 23 and 24

26. Repeat step 22

27. Place the tubes under the fume hood for 3 minutes to dry the pellet

28. Add 100 ul of TE buffer to each tube to resuspend the pellet.

If the pellet does not easily resuspend, heat to 30°C and gently agitate by hand. TE buffer was autoclaved.

<u>DNA purification and isolation</u>

29. Pool the two samples together in fresh 1.5 ml Eppendorf tubes

30. Add 0.2ul of DNase free RNase

31. Incubate at 37°C for 15 minutes

32. Add 20ul of Proteinase K and 200 ul of Buffer AL

33. Incubate at 70°C for 10 minutes

34. Add 200 ul of 100% ethanol and mix gently

35. Transfer the samples into spin columns

36. Centrifuge at 16000 x g for 1 minute at room temperature

37. Discard flow through

38. Add 500 ul Buffer AW 1 and repeat 36 and 37

39. Add 500 ul Buffer AW2 and repeat 36 and 37

40. Repeat 36

41. Transfer columns into fresh collection tubes

42. Add 200 ul of elution buffer AE

43. Leave at room temperature for 2 minutes

44. Repeat 36

45. Aliquot 50ul of the eluted DNA into 3 fresh 1.5 ml Eppendorf tubes

46. Use 2ul of sample to test DNA yield and purity using DeNovix 11

47. Record sample concentration and purity

48. Store 1 aliquot in a box ready to be labelled and transferred for PCR and sequencing at -20°C

49. Store 2 aliquots in a separate box as duplicates for back up.

1. Results

3.1. Read depth was most influenced by DNA yield and individual

Table S1. Model specification for alpha diversity metric Gamm selection

Model	Term				
	treatment	timepoint	treatment*timepoint	group	group*treatment
1	X	X	X	X	0
2	X	X	X	0	0
3	X	X	0	X	0
4	X	0	0	X	0
5	0	X	0	X	0
6	X	X	0	0	0
7	X	0	0	0	0
8	0	X	0	0	0
9	0	0	0	X	0
10	X	X	0	X	X
11	X	0	0	X	X

Table S2. Faith's PD Gamm selection

	K	LOGLIK	AICC	delta_AICC	omega
model1	2	-399.1370	804.2740	427.91444	1.200971e-93
model2	26	-187.1730	406.3459	29.98633	3.079821e-07
model3	24	-200.6898	429.3796	53.02007	3.067696e-12
model4	22	-187.5668	399.1335	22.77392	1.134172e-05
model5	21	-188.0102	398.0203	21.66077	1.978781e-05
model6	18	-191.6094	399.2189	22.85930	1.086776e-05
model7	20	-201.1066	422.2132	45.85366	1.104023e-10
model8	19	-201.4824	420.9648	44.60521	2.060987e-10
model9	16	-205.2129	422.4257	46.06616	9.927373e-11
model10	17	-192.0422	398.0844	21.72480	1.916432e-05
model11	29	-169.1798	376.3596	0.00000	9.999385e-01

Table S3. Observed diversity Gamm selection

	K	LOGLIK	AICC	delta_AICC	omega
model1	2	-398.7398	803.4796	439.66404	3.374145e-96
model2	26	-182.9695	397.9390	34.12343	3.892145e-08
model3	24	-199.5814	427.1627	63.34713	1.755261e-14
model4	22	-183.3618	390.7236	26.90803	1.435467e-06
model5	21	-183.9801	389.9601	26.14456	2.102706e-06
model6	18	-187.2035	390.4070	26.59147	1.681639e-06
model7	20	-199.9863	419.9726	56.15708	6.392076e-13
model8	19	-200.5216	419.0431	55.22754	1.017391e-12
model9	16	-203.6547	419.3093	55.49374	8.906018e-13
model10	17	-187.8125	389.6250	25.80940	2.486319e-06

model11	29	-162.9078	363.8156	0.00000	9.999923e-01

Table S4. Shannon's Diversity Gamm selection

	K	LOGLIK	AICC	delta_AICC	omega
model1	2	-400.95622	807.9124	640.42339	8.586670e-140
model2	26	-83.96670	199.9334	32.44434	9.011485e-08
model3	24	-99.44630	226.8926	59.40353	1.260908e-13
model4	22	-84.43396	192.8679	25.37887	3.083509e-06
model5	21	-84.86144	191.7229	24.23381	5.466275e-06
model6	18	-94.40430	204.8086	37.31954	7.873378e-09
model7	20	-99.87102	219.7420	52.25297	4.502027e-12
model8	19	-100.26048	218.5210	51.03190	8.290087e-12
model9	16	-109.65383	231.3077	63.81860	1.386640e-14
model10	17	-94.84385	203.6877	36.19864	1.378989e-08
model11	29	-64.74453	167.4891	0.00000	9.999913e-01

Faith's Phylogenetic Diversity

Table S5: Parametric coefficients for faiths phylogenetic diversity scaled as predicted by the Group and Treatment factors

	Estimate	Std.Error	T value	Pr(>|t|)
(Intercept)	0.26302	0.22891	1.149	0.25178
Group2	0.06475	0.31257	0.207	0.83608
Group1	-0.78135	0.28670	-2.725	0.00693**
TreatmentCOLD	-0.39082	0.21761	-1.796	0.07384.
TreatmentCLX	-0.25564	0.21619	-1.182	0.23826
TreatmentNAP	-0.33083	0.21718	-1.523	0.12908
TreatmentFTA	-0.54425	0.22001	-2.474	0.01411*
Group2:TreatmentCOLD	0.27650	0.30659	0.902	0.36810
Group1:TreatmentCOLD	0.50534	0.30722	1.645	0.10139
Group2:TreatmentCLX	0.47131	0.30578	1.541	0.12463
Group1:TreatmentCLX	0.47391	0.30610	1.548	0.12297
Group2:TreatmentNAP	0.29934	0.30602	0.978	0.32902
Group1:TreatmentNAP	0.38897	0.30664	1.268	0.20593
Group2:TreatmentFTA	0.97742	0.31028	3.150	0.00185**
Group1:TreatmentFTA	0.38768	0.31033	1.249	0.21287

Approximate significance of smooth terms:

edf Ref.df F p-value

s(total_reads) 1.000 1 617.293 < 2e-16 ***

s(Pony) 6.219 8 4.486 7.85e-06 ***

R-sq.(adj) = 0.813

Scale est. = 0.17564   n = 248

Observed Diversity

Table S6: Parametric coefficients for observed diversity scaled as predicted by the Group and Treatment factors

	Estimate	Std.Error	tvalue	Pr(>|t|)
(Intercept)	0.2409	0.2265	1.064	0.28864
Group2	0.1186	0.3093	0.384	0.70164
Group1	-0.8069	0.2845	-2.836	0.00498**
TreatmentCOLD	-0.3258	0.2161	-1.508	0.13297
TreatmentCLX	-0.2286	0.2146	-1.065	0.28798
TreatmentNAP	-0.2881	0.2156	-1.336	0.18282
TreatmentFTA	-0.5747	0.2184	-2.631	0.00910**
Group2:TreatmentCOLD	0.1802	0.3044	0.592	0.55438
Group1:TreatmentCOLD	0.4751	0.3050	1.558	0.12071
Group2:TreatmentCLX	0.4035	0.3036	1.329	0.18509
Group1:TreatmentCLX	0.4944	0.3039	1.627	0.10515
Group2:TreatmentNAP	0.2436	0.3038	0.802	0.42345
Group1:TreatmentNAP	0.4095	0.3044	1.345	0.17995
Group2:TreatmentFTA	0.9913	0.3081	3.218	0.00148**
Group1:TreatmentFTA	0.4643	0.3081	1.507	0.13324

Approximate significance of smooth terms:

edf Ref.df F p-value

s(total_reads) 1.000 1 616.377 < 2e-16 ***

s(Pony) 6.165 8 4.321 1.21e-05 ***

R-sq.(adj) = 0.816

Scale est. = 0.17313   n = 248

Shannon's Diversity

Table S7: Parametric coefficients for Shannon's diversity scaled as predicted by the Group and Treatment factors

	Estimate	Std.Error	tvalue	Pr(>|t|)
(Intercept)	0.121702	0.167097	0.728	0.46717

Group2	0.375022	0.227833	1.646	0.10116
Group1	-0.468781	0.206379	-2.271	0.02407*
TreatmentCOLD	-0.147596	0.156608	-0.942	0.34698
TreatmentCLX	-0.161064	0.155540	-1.036	0.30155
TreatmentNAP	-0.149221	0.156127	-0.956	0.34022
TreatmentFTA	-0.460304	0.159793	-2.881	0.00436**
Group2:TreatmentCOLD	0.011436	0.220514	0.052	0.95869
Group1:TreatmentCOLD	0.320381	0.220847	1.451	0.14827
Group2:TreatmentCLX	0.064262	0.220058	0.292	0.77054
Group1:TreatmentCLX	0.335700	0.220371	1.523	0.12909
Group2:TreatmentNAP	0.004866	0.219908	0.022	0.98237
Group1:TreatmentNAP	0.263374	0.220439	1.195	0.23345
Group2:TreatmentFTA	0.512656	0.223719	2.292	0.02287*
Group1:TreatmentFTA	0.213549	0.225252	0.948	0.34413

Approximate significance of smooth terms:

edf Ref.df F p-value

s(total_reads) 3.331 3.331 424.024 < 2e-16 ***

s(Pony) 6.397 8.000 5.156 1.83e-06 ***

R-sq.(adj) = 0.904

Scale est. = 0.090643 n = 248

3.4 Microbial community was distinct in FTA cards

Table S8. Relative abundance of the dominant taxa averaged across all samples

Taxonomy	Relative abundance (%)
Phylum	
Firmicutes	61.27
Bacteroidetes	27.02
Spirochaetes	5.15
Firbobacterota	3.97
Proteobacteria	1.00
Verrucomicrobia	0.85
Actinobacteriota	0.36

Patescibacteria	0.17
Synergistota	0.11
Planctomyecetota	0.04
Chlorflexi	0.04
Desulphobacterota	0.02
Elusimicrobiota	0.01
Myxococcota	<0.01
Class (Top 20)	
Clostridia	52.55
Bacteroidia	27.02
Bacilli	6.42
Spirochaetia	5.15
Fibrobacteria	3.97
Negativicutes	2.30
Gammaproteobacteria	1.00
Verrucomicrobiae	0.78
Coriobacteriia	0.32
Saccharimonadia	0.19
Syndergistia	0.11
Lentisphaeria	0.07
Alphaproteobacteria	0.05
Planctomycetes	0.04
Actinobacteria	0.04
Anaerolineae	0.04
Desulfovibrionia	0.02

Endomicrobia	0.01
Myxococcia	<0.01
Order (Top 30)	
Lachnospirales	29.61
Bacteroidales	27.00
Oscillospirales	17.38
Spirochaetales	5.15
Fibrobacterales	3.97
Erysipelotrichales	2.96
Christensenellales	2.56
Peptostreptococcales-Tissierellales	1.95
Acidaminococcales	1.86
Lactobacillales	1.79
Bacillales	1.30
Verrucomicrobiales	0.78
Clostridiales	0.77
Pseudomonadales	0.61
Veillonellales-Selenomonadales	0.44
Coriobacteriales	0.32
Mycoplasmatales	0.27
Eubacteriales	0.24
Enterobacterales	0.21
Saccharimonadales	0.19
Synergistales	0.11
Burkholderiales	0.09

Acholeplasmatales	0.06
Rickettsiales	0.05
Paenibacillales	0.04
Pirellulales	0.04
Anaerolineales	0.04
Oligosphaerales	0.04
Micrococcales	0.04
Monoglobales	0.04
Family (Top 30)	
Oscillospiraceae	8.79
p-251-o5	7.85
Rikenellaceae	5.82
Spirochaetaceae	5.15
Prevotellaceae	5.13
Fibrobacteraceae	3.97
Ruminococcaceae	3.89
Christensenellaceae	2.56
Erysipelatoclostridiaceae	2.43
Bacteroidales RF16 group	2.31
Anaerovoracaceae	1.94
[Eubacterium] coprostanoligenes group	1.89
Acidaminococcaceae	1.86
F082	1.85
Bacteroidales UCG-001	1.49
UCG-010	1.44

Bacteroidales BS11 gut group	1.38
Hungateiclostridiaceae	1.35
Streptococcaceae	1.32
Planococcaceae	1.08
Akkermansiaceae	0.78
Clostridiaceae	0.77
Paludibacteraceae	0.62
Erysipelotrichaceae	0.53
Selenomonadaceae	0.44
Moraxellaceae	0.40
Mycoplasmataceae	0.27
Eubacteriaceae	0.23
Carnobacteriaceae	0.22
Genus	
Lachnospiraceae AC2044 group	10.46
Treponema	5.03
Rikenellaceae RC9 gut group	4.46
Lachnospiraceae UCG-009	4.24
Fibrobacter	3.97
Ruminococcus	3.27
Lachnospiraceae XPB1014 group	3.26
NK4A214 group	3.25
Christensenellaceae R-7 group	2.54
UCG-004	2.42
Prevotella	2.16

UCG-005	2.06
Phascolarctobacterium	1.35
Saccharofermentans	1.32
Streptococcus	1.32
UCG-002	1.21
hoa5-07d05 gut group	0.90
Prevotellaceae UCG-001	0.87
Akkermansia	0.78
Mogibacterium	0.71
Oribacterium	0.69
Clostridium sensu stricto 1	0.68
Prevotellaceae UCG-003	0.66
Anaerovorax	0.50
Lachnospiraceae NK4A136 group	0.48
Family XIII AD3011 group	0.46
XBB1006	0.46
Alloprevotella	0.45
Solibacillus	0.42
Acinetobacter	0.40

Table 9. Relative abundance of top 10 taxa averaged by treatment at the Phylum, Class, Order, Family and Genus levels.

Phylum	Taxa	R. Abund (%)	Taxa	R. Abund (%)	Taxa	R. Abund (%)	Taxa	R. Abund (%)	Taxa	R. Abund (%)
	- 80 REF		COLD		CLX		NAP		FTA	
Phylum	Firmicutes	55.90	Firmicutes	58.97	Firmicutes	62.20	Firmicutes	54.77	Firmicutes	75.96
	Bacteroidota	32.31	Bacteroidota	28.09	Bacteroidota	24.58	Bacteroidota	31.62	Bacteroidota	17.55
	Spirochaetota	5.58	Spirochaetota	5.79	Spirochaetota	6.11	Spirochaetota	6.69	Fibrobacterota	2.14
	Fibrobacterota	5.04	Fibrobacterota	3.49	Fibrobacterota	3.39	Fibrobacterota	5.59	Verrucomicrobiota	1.35
	Verrucomicrobiota	0.65	Proteobacteria	2.28	Proteobacteria	2.23	Verrucomicrobiota	0.70	Actinobacteriota	1.22
	Patescibacteria	0.13	Verrucomicrobiota	0.73	Verrucomicrobiota	0.85	Proteobacteria	0.18	Spirochaetota	1.17
	Actinobacteriota	0.13	Actinobacteriota	0.21	Patescibacteria	0.27	Patescibacteria	0.16	Synergistota	0.17
	Synergistota	0.09	Patescibacteria	0.25	Actinobacteriota	0.18	Actinobacteriota	0.14	Patescibacteria	0.12
	Planctomycetota	0.05	Synergistota	0.11	Synergistota	0.12	Synergistota	0.06	Chloroflexi	0.11
	Proteobacteria	0.04	Planctomycetota	0.03	Chloroflexi	0.03	Planctomycetota	0.04	Planctomycetota	0.09
Class	Clostridia	48.67	Clostridia	45.37	Clostridia	53.04	Clostridia	47.86	Clostridia	69.39
	Bacteroidia	32.31	Bacteroidia	28.09	Bacteroidia	24.58	Bacteroidia	31.62	Bacteroidia	17.55
	Spirochaetia	5.58	Bacilli	11.56	Bacilli	6.42	Spirochaetia	6.69	Bacilli	4.37
	Bacilli	5.06	Spirochaetia	5.79	Spirochaetia	6.11	Fibrobacteria	5.59	Negativicutes	2.20
	Fibrobacteria	5.04	Fibrobacteria	3.49	Fibrobacteria	3.39	Bacilli	4.59	Fibrobacteria	2.14
	Negativicutes	2.17	Gammaproteobacteria	2.26	Negativicutes	2.74	Negativicutes	2.31	Verrucomicrobiae	1.29
	Verrucomicrobiae	0.57	Negativicutes	2.04	Gammaproteobacteria	2.13	Verrucomicrobiae	0.62	Coriobacteriia	1.22
	Saccharimonadia	0.13	Verrucomicrobiae	0.68	Verrucomicrobiae	0.79	Saccharimonadia	0.16	Spirochaetia	1.17
	Coriobacteriia	0.13	Saccharimonadia	0.25	Saccharimonadia	0.27	Coriobacteriia	0.13	Synergistia	0.17
	Synergistia	0.09	Actinobacteria	0.14	Coriobacteriia	0.13	Gammaproteobacteria	0.10	Saccharimonadia	0.12
Order	Bacteroidales	32.31	Bacteroidales	28.07	Lachnospirales	29.66	Bacteroidales	31.60	Lachnospirales	36.22
	Lachnospirales	28.75	Lachnospirales	26.34	Bacteroidales	24.56	Lachnospirales	27.70	Oscillospirales	23.20
	Oscillospirales	16.39	Oscillospirales	13.12	Oscillospirales	17.42	Oscillospirales	17.26	Bacteroidales	17.55

	Spirochaetales	5.58	Spirochaetales	5.79	Spirochaetales	6.11	Spirochaetales	6.69	Peptostreptococcales-Tissierellales	4.82
	Fibrobacterales	5.04	Bacillales	4.97	Fibrobacterales	3.39	Fibrobacterales	5.59	Christensenellales	3.78
	Erysipelotrichales	3.49	Fibrobacterales	3.49	Erysipelotrichales	2.67	Erysipelotrichales	2.72	Erysipelotrichales	2.74
	Christensenellales	2.06	Erysipelotrichales	3.17	Christensenellales	2.49	Christensenellales	2.05	Fibrobacterales	2.14
	Acidaminococcales	1.88	Lactobacillales	2.81	Acidaminococcales	2.41	Acidaminococcales	1.97	Lactobacillales	1.39
	Peptostreptococcales-Tissierellales	1.40	Christensenellales	2.53	Lactobacillales	2.32	Lactobacillales	1.19	Verrucomicrobiales	1.29
	Lactobacillales	1.23	Clostridiales	1.92	Clostridiales	1.77	Peptostreptococcales-Tissierellales	0.79	Coriobacteriales	1.22
Family	*Lachnospiraceae*	28.74	*Lachnospiraceae*	26.32	*Lachnospiraceae*	29.64	*Lachnospiraceae*	27.66	*Lachnospiraceae*	36.21
	p-251-o5	9.93	*p-251-o5*	7.76	*Oscillospiraceae*	9.40	*p-251-o5*	9.72	*Oscillospiraceae*	12.23
	Oscillospiraceae	7.67	*Oscillospiraceae*	7.53	*p-251-o5*	7.86	*Oscillospiraceae*	7.42	*Ruminococcaceae*	5.70
	Rikenellaceae	6.61	*Rikenellaceae*	6.66	*Spirochaetaceae*	6.11	*Spirochaetaceae*	6.69	*Anaerovoracaceae*	4.82
	Prevotellaceae	5.99	*Spirochaetaceae*	5.79	*Rikenellaceae*	5.88	*Rikenellaceae*	6.28	*Prevotellaceae*	4.15
	Spirochaetaceae	5.58	*Prevotellaceae*	4.94	*Prevotellaceae*	4.22	*Prevotellaceae*	6.25	*Christensenellaceae*	3.78
	Fibrobacteraceae	5.04	*Planococcaceae*	4.02	*Ruminococcaceae*	3.70	*Fibrobacteraceae*	5.59	*p-251-o5*	3.55
	Ruminococcaceae	3.77	*Fibrobacteraceae*	3.49	*Fibrobacteraceae*	3.39	*Ruminococcaceae*	4.46	*Rikenellaceae*	3.46
	Erysipelatoclostridiaceae	2.95	*Erysipelatoclostridiaceae*	2.71	*Christensenellaceae*	2.49	*Bacteroidales RF16 group*	2.64	*[Eubacterium] coprostanoligenes group*	2.48
	Bacteroidales RF16 group	2.73	*Bacteroidales RF16 group*	2.57	*Acidaminococcaceae*	2.41	*Erysipelatoclostridiaceae*	2.34	*Fibrobacteraceae*	2.14
Genus	*Lachnospiraceae AC2044 group*	10.81	*Lachnospiraceae AC2044 group*	8.25	*Lachnospiraceae AC2044 group*	10.58	*Lachnospiraceae AC2044 group*	10.05	*Lachnospiraceae AC2044 group*	12.81
	Treponema	5.46	*Treponema*	5.63	*Treponema*	6.01	*Treponema*	6.56	*Lachnospiraceae XPB1014 group*	7.01
	Rikenellaceae RC9 gut group	5.14	*Rikenellaceae RC9 gut group*	5.09	*Lachnospiraceae UCG-009*	5.02	*Fibrobacter*	5.59	*NK4A214 group*	5.83
	Fibrobacter	5.04	*Lachnospiraceae UCG-009*	4.82	*Rikenellaceae RC9 gut group*	4.42	*Lachnospiraceae UCG-009*	4.98	*Ruminococcus*	4.82
	Lachnospiraceae UCG-009	4.37	*Fibrobacter*	3.49	*Fibrobacter*	3.39	*Rikenellaceae RC9 gut group*	4.63	*Christensenellaceae R-7 group*	3.74

Ruminococcus	3.23	*UCG-004*	2.71	*Ruminococcus*	3.10	*Ruminococcus*	3.93	*Mogibacterium*	2.97
UCG-004	2.94	*NK4A214 group*	2.71	*NK4A214 group*	3.08	*Prevotella*	2.79	*Rikenellaceae RC9 gut group*	2.89
Prevotella	2.67	*Christensenellaceae R-7 group*	2.52	*Lachnospiraceae XPB1014 group*	2.82	*Lachnospiraceae XPB1014 group*	2.50	*Fibrobacter*	2.14
NK4A214 group	2.44	*Lachnospiraceae XPB1014 group*	2.36	*UCG-005*	2.70	*NK4A214 group*	2.47	*UCG-004*	2.12
Christensenellaceae R-7 group	2.05	*Prevotella*	2.06	*Christensenellaceae R-7 group*	2.49	*UCG-004*	2.33	*Lachnospiraceae UCG-009*	1.80

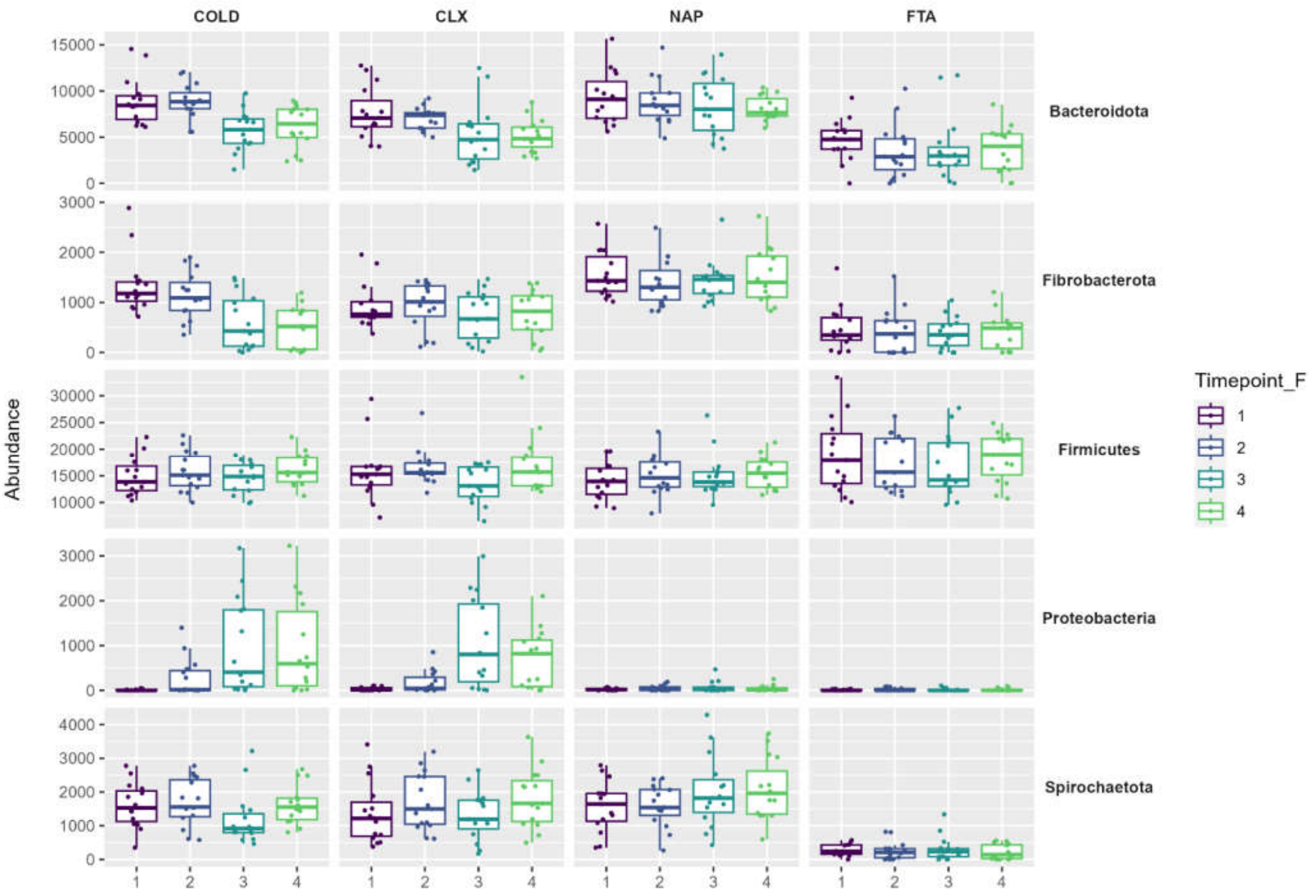

Supplementary Figure S1. Raw abundance of the most dominant phyla in equine faecal samples over time at room temperature under different preservation treatments. The X axis shows measurements taken at timepoint 1 (0 hours at room temperature), timepoint 2 (24 hours at room temperature), timepoint 3 (72 hours at room temperature) and timepoint 4 (150 hours at room temperature), and corresponding treatment is indicated in bold at the top of the plot.
